# Post-Translational Modifications of TRP Channels

**DOI:** 10.3390/cells3020258

**Published:** 2014-04-08

**Authors:** Olaf Voolstra, Armin Huber

**Affiliations:** Department of Biosensorics, Institute of Physiology, Universität Hohenheim, 70599 Stuttgart, Germany; E-Mail: armin.huber@uni-hohenheim.de

**Keywords:** post-translational modification, TRP channels, N-linked glycosylation, covalent modification of cysteines, phosphorylation

## Abstract

Transient receptor potential (TRP) channels constitute an ancient family of cation channels that have been found in many eukaryotic organisms from yeast to human. TRP channels exert a multitude of physiological functions ranging from Ca^2+^ homeostasis in the kidney to pain reception and vision. These channels are activated by a wide range of stimuli and undergo covalent post-translational modifications that affect and modulate their subcellular targeting, their biophysical properties, or channel gating. These modifications include N-linked glycosylation, protein phosphorylation, and covalent attachment of chemicals that reversibly bind to specific cysteine residues. The latter modification represents an unusual activation mechanism of ligand-gated ion channels that is in contrast to the lock-and-key paradigm of receptor activation by its agonists. In this review, we summarize the post-translational modifications identified on TRP channels and, when available, explain their physiological role.

## 1. Transient Receptor Potential Channels

The first transient receptor potential (TRP) channel was uncovered in the compound eye of *Drosophila melanogaster*. A *Drosophila* mutant was isolated that showed a transient electrical light response in electroretinographic recordings upon application of a prolonged light stimulus [1]. The name transient receptor potential for this mutant was coined in 1975 by Minke and colleagues [2]. The corresponding TRP gene was cloned by Montell and collegues [3] and the TRP protein was first suggested to be a Ca^2+^ permeable ion channel by Hardie and Minke [4]. Later, it became obvious that TRP channels constitute a large family of cation channels that have been found in many eukaryotic organisms from yeast to human. TRP channels serve a multitude of functions ranging from sensory functions such as pain reception and vision to Ca^2+^ homeostasis. TRP channels exhibit considerable sequence homology and share six predicted transmembrane regions and intracellular N- and C-termini. The *Drosophila* TRP channel belongs to the subfamily of TRPC (canonical) channels. The TRPV (vanniloid) and TRPM (melastatin) subfamilies display the strongest similarity to TRPC channels. Other subfamilies encompass the TRPN (NOMPC-like), TRPA (ankyrin transmembrane proteins), TRPML (mucolipin), and TRPP (polycystin) channels. The channel pore is mainly formed by a pore-forming loop between the fifth and sixth transmembrane domain upon tetramerization of TRP subunits. Recently, the structure of TRPV1 has been resolved to 3.4 Å resolution by single particle cryo-electron microscopy [5,6]. These studies revealed a channel pore with a dual gating mechanism composed of a selectivity filter formed by the S5-S6 pore loop, that is located near the outer surface of the channel, and a second lower gate formed by parts of the S6 helix. Both gates are allosterically coupled. Agonists like the spider toxin DkTx bind close to and activate the upper gate while the hydrophobic agonists capsaicine and resiniferatoxin bind to and activate the lower gate deeper within the membrane. In general, activation of TRP channels can either occur via a receptor and a signaling cascade that finally culminates in the opening of the channel, which is the canonical activation mechanism for TRPC channels, or the channel itself is a receptor as exemplified by TRPV1.

## 2. The Various Types of Post-Translational Modifications

Post-translational modification of proteins is defined as the processing of a protein during or after biosynthesis. Protein processing comprises regulated proteolysis of the polypeptide chain, attachment of coenzymes such as heme groups and covalent modifications of amino acid residues. The latter will be the focus of this review. Post-translational modifications largely enhance the flexibility and variability of an organism’s proteome, that is it allows the generation of a huge number of different proteins from a relatively limited pool of genes. In addition, many post-translational modifications are reversible and have a regulatory role. This includes regulation of the subcellular localization of proteins and control of protein-protein interactions. Post-translational modifications may also affect protein stability or regulate the activity of enzymes and ion channels. The same protein can undergo various post-translational modifications that may have opposite effects on protein function (see for example phosphorylation of TRPV1, Section 6). The post-translational modifications of TRP channels that are discussed in this review are summarized in Table 1.

**Table 1:** Post-translational modifications of TRP channels. * partially as proposed by [7].

Channel	Function *	Modification	Modified Site	Location of Modified Site	Modification Regulates	Reference
TRPA1 (see Figure 1A)	thermo-sensation (noxious cold), chemo-sensation, nociception, O_2_ sensing	covalent modification by electrophiles	C621 C641 C665	N-terminus	channel gating	[8]
		covalent modification by electrophiles	C414 C421 C621			[9]
		covalent modification by inflammatory mediators	C421 C621			[10]
		hydroxylation	P394			[11]
		oxidation	C633			
			C856	2nd intracellular loop		
TRPC3	BDNF-signaling in the brain	N-linked glycosylation	N418	1st extracellular loop	channel activity (by surface expression?)	[12]
		phosphorylation by PKC	T646 S712	2nd intracellular loop	channel gating	[13,14]
		phosphorylation by PKG	T11 S263	N-terminus		[15]
		phosphorylation by Src kinase				[16]
TRPC5	brain development	S-nitrosylation	C553 C558	adjacent to pore-forming loop	channel gating	[17]
		phosphorylation by PKC	T970			[18]
TRPC6	signaling in smooth muscle	N-linked glycosylation	N473 N561	1st and 2nd extracellular loop	channel activity (by surface expression?)	[12]
		phosphorylation by PKC			channel inhibition	[19]
		phosphorylation by Src family kinase Fyn	T970	C-terminus	carbachol-mediated desensitization	[20]
		phosphorylation by CaMKII			channel activation	[21]
TRPM4b	regulation of Ca^2+^ entry into the cell	N-linked glycosylation	N988	adjacent to pore-forming loop	surface expression	[22]
TRPM7	Mg^2+^ homeostasis and reabsorption in kidney and intestine, cell migration	autophorylation	several sites	C-terminus	substrate recognition	[23]
TRPM8	thermo-sensation (cold), sperm motility, acrosome reaction	N-linked glycosylation	N934	adjacent to pore-forming loop	response to cold and menthol	[24,25,26]
		Polyester modification	several sites	N-terminus and S3-S4 linker	channel function	[27]
TRPV1 (see Figure 1B)	thermo-sensation (heat), nociception	N-linked glycosylation	N604	adjacent to pore-forming loop	ligand binding or gating properties	[28,29]
		cysteine modification	C158	N-terminus	activation by cysteine-modifying compounds	[30]
		cysteine modification	C158 C387 C391		sensitization by oxidative stress	[31]
			C767	C-terminus		
		phosphorylation by PKC	S502	1st intracellular loop	potentiation	[32]
			S801	C-terminus		
		phosphorylation by PKA	S117	N-terminus	prevention of desensitization	[33]
			T145 T371		sensitization	[34]
			S502	1st intracellular loop		
		phosphorylation by c-Src	Y200	N-terminus	surface expression	[35]
		phosphorylation by CaMKII	S502	1st intracellular loop	channel activity	[36]
			T705	C-terminus		
TRPV2	thermo-sensation (noxious heat), nociception	N-linked glycosylation	N570 (alignment)	adjacent to pore-forming loop		[37,38]
TRPV4	tonicity sensing	N-linked glycosylation	N651	adjacent to pore-forming loop	channel activity (through surface expression?)	[39]
		phosphorylation by Src	Y253	N-terminus	channel activity	[39,40]
TRPV5	Ca^2+^ reabsorption in kidney	N-linked glycosylation	N358	1st extracellular loop	surface expression	[41]
TRPV6	Ca^2+^ reabsorption in intestine	N-linked glycosylation	N357	1st extracellular loop	surface expression	
dTRP (see Figure 2A)	generation of the photoreceptor potential	phosphorylation	S15	N-terminus		[42,43]
			S717 S721	C-terminus		
			S726 S828			
			T849 T864			
			S867 S872			
			S875 S876			
			S881 S884			
			S936 S956			
			S958 S961			
			T963 S964			
			S982 S990			
			T998 T1036			
			T1049 S1056			
			S1123 S1253 S1254			
dTRPL (see Figure 2B)	generation of the photoreceptor potential	phosphorylation	S20	N-terminus		[44]
			S730 S927	C-terminus	channel stability	
			S931 T989			
			S1000 T1114			
			S1115 S1116			

**Figure 1:**
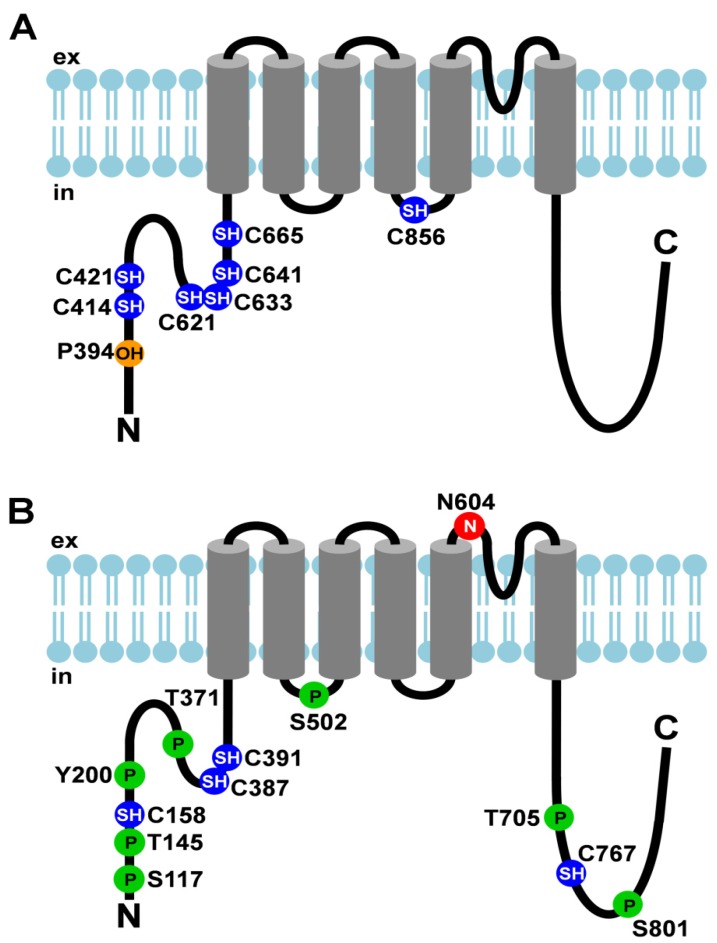
Post-translational modifications of TRPA1 and TRPV1. (**A**) Cartoon depicting TRPA1 and its post-translational modifications; (**B**) Cartoon depicting TRPV1 and its post-translational modifications. Amino acid residues that undergo post- translational modifications are depicted as circles. The N-linked glycosylation site is shown as a red circle with an N, cysteines that are modified are shown as blue circles with SH, hydroxylated proline is depicted as an orange circle with OH, and phosphorylation sites are represented by green circles with P. ex, extracellular; in, intracellular.

## 3. N-Linked Glycosylation of TRP Channels

Glycosylation is the enzymatically catalyzed covalent addition of sugars to lipids or proteins. Proteins can be glycosylated at the hydroxyl group of Ser and Thr residues (O-linked glycosylation) or at the amino group of Asn residues (N-linked glycosylation) that are part of an Asn-X-Ser/Thr consensus sequence whereby X can be every amino acid but is never a Pro residue. N-linked glycosylation is the prevalent covalent modification of eukaryotic proteins and serves many functions. It is involved in subcellular targeting of proteins, the protection of proteins against denaturation and proteolysis, affects protein turnover, influences the charge and the isoelectric point of proteins, promotes rigidity of proteins, and helps membrane proteins to take up the proper orientation within the bilayer [45,46].

For N-linked glycosylation, a generic oligosaccharide consisting of 14 sugars (two N acetylglucosamines, nine mannoses, and three glucoses) is synthesized at the ER membrane. The monosaccharide building blocks are successively added to dolichyl pyrophosphate, a lipid carrier residing within the ER membrane. Upon completion of this oligosaccharide, it is transferred en bloc to a nascent target protein by oligosaccharyl transferase that recognizes the Asn-X-Ser/Thr amino acid motif. Subsequently, the oligosaccharide is trimmed leaving a core oligosaccharide composed of two N-acetylglucosamines and three mannose residues. To achieve the vast variety of glycosylation patterns, the core oligosaccharides are altered by glycosyltransferases and glycosidases in the endoplasmic reticulum and in the Golgi complex. While glycosyltransferases catalyze the addition of a sugar to a specific oligosaccharide, glycosidases promote the hydrolyzation of certain sugars from a specific oligosaccharide. The addition or removal of a certain monosaccharide generates the substrate for the next enzyme that specifically recognizes its substrate to catalyze the addition or removal of another monosaccharide. Mammalian cells resort to nine different monosaccharide building blocks [45].

An example of a TRP channel that is modified by N-linked glycosylation is the vanniloid receptor subtype 5 (TRPV5). TRPV5 is expressed in the distal convoluted tubules of the kidney where it facilitates Ca^2+^ uptake from the glomerular filtrate on the apical extracellular side into the cell. Interestingly, TRPV5 is among the TRP channels with the highest Ca^2+^ selectivity. Within the cell, Ca^2+^ binds to calbindin-D_28K_ and by diffusion reaches the basolateral membrane where it is released into blood vessels. Consistently, *TRPV5* knockout mice display impaired renal Ca^2+^ reabsorption and reduced bone thickness [47]. The TRPV5 knockout mice were used by Chang and co-workers in an attempt to identify proteins that are involved in Ca^2+^ homeostasis [41]. In *TRPV5* knockout mice, the expression of a gene product called Klotho was diminished. In the wild type, Klotho was abundantly expressed in the kidney. At that time, it was already known that an insertional mutation of the *klotho* gene led to a compilation of phenotypes reminiscent of aging, including a short lifespan, arteriosclerosis, osteoporosis, and infertility that were observed in the mouse [48]. When Chang and colleagues co-expressed mouse TRPV5 and KLOTHO in human embryonic kidney (HEK 293) cells, they observed an increase in cellular Ca^2+^ uptake. Since KLOTHO had been detected in extracellular liquids such as urine, serum, and cerebrospinal fluid [49], they reasoned that KLOTHO might regulate TRPV5 activity from the extracellular side of the cell. Therefore, they treated TRPV5-expressing cells with KLOTHO-containing supernatant from KLOTHO-expressing cells. Indeed, application of extracellular KLOTHO also increased TRPV5 channel activity. KLOTHO had been shown to exhibit β-glucuronidase activity [50]. Consistently, β-glucuronidase treatment of TRPV5-expressing cells mimicked the effect of incubation with KLOTHO-containing supernatant. Abolition of a predicted N glycosylation site, Asn358, by exchange to Gln prevented KLOTHO-induced increase of TRPV5 channel activity. Finally, KLOTHO-mediated rise in channel activity could be attributed to an increased expression of TRPV5 at the plasma membrane [41]. Taken together, the authors concluded that the β-glucuronidase KLOTHO hydrolyzes the extracellular glycan attached to TRPV5 and thereby traps the ion channel in the apical membrane in the distal nephron. Imbalances of this process have deleterious effects as exemplified by the *klotho* mutant mouse. Notably, TRPV6 that is believed to facilitate intestinal Ca^2+^ resorption is highly homologous to TRPV5. Presence of KLOTHO also increased the activity of TRPV6 and mutation of a predicted N glycosylation site (exchange of Asn357 to Gln357) within the first extracellular loop abolished the KLOTHO-mediated increase in activity [41]. Together with the fact that KLOTHO is present in extracellular fluids, it is likely that KLOTHO regulates the activity of other channels by cleavage of extracellular glycans. Recently, it has been shown that tissue transglutaminase cross-links the N-glycosylated fraction of TRPV5 leading to its inactivation probably by structural changes that reduce the pore diameter [51]. This finding points to a complex regulation of TRPV5.

Vannier and colleagues set out to map the membrane topology of human TRPC3 by exploiting the fact that N-linked glycosylation occurs exclusively at sites that are exposed to the extracellular side [52]. *En passant*, they identified Asn418 residing within the first extracellular loop as an endogenous TRPC3 glycosylation site. Through introduction of glycosylation sites by site directed mutagenesis and assessment of the glycosylation patterns of the resulting mutant TRPC3, the authors were able to generate a map of the transmembrane topology of TRPC3. As a result, intracellular localization of the N- and C-termini and the existence of six transmembrane domains could be verified but the first of seven predicted regions with the potential to span the membrane was shown to be located within the cytosol. Besides glycosylation of the first extracellular loop, TRPC6 is additionally glycosylated at the second extracellular loop [12]. While TRPC3 exhibits constitutive channel activity, TRPC6 activity is tightly regulated by diacylglycerol. However, by removal of the second glycosylation site, TRPC6 could be converted to a constitutively active channel. Reversely, introduction of a second N-glycosylation site to TRPC3 in the second extracellular loop led to a reduction of TRPC3 basal activity [12]. These results suggest that glycosylation of TRPC3 and TRPC6 can profoundly affect channel activity.

Besides glycosylation of the first and second extracellular loop, N-linked glycosylation at the pore-forming third extracellular loop between the fifth and sixth transmembrane helix has been reported for TRPV1, TRPV4, TRPM8, and TRPM4b [22,24,28,29,37,38,39,40]. TRPV1 is expressed in a subset of nociceptive neurons. Activation of TRPV1 by noxious heat, by spider toxins, or by capsaicin, the pungent ingredient of chili peppers ultimately leads to the perception of pain in mammals. Therefore, TRPV1 is a polymodal detector of pain-inducing chemical and physical stimuli. By identification of possible N-linked glycosylation sites and subsequent topology prediction, Jahnel and colleagues identified Asn604 as a putative N-linked glycosylation site of the rat TRPV1 channel [28]. Mutation of Asn604 resulted in absence of TRPV1 glycosylation demonstrating that this is the only glycosylation site. When Wirkner and coworkers expressed TRPV1 in which the Asn604 glycosylation site was ablated by exchange to Thr, they observed a decreased sensitivity towards capsaicin and thus concluded that glycosylation might regulate ligand binding or gating properties of TRPV1 [29]. However, since the three-dimensional structure of TRPV1 revealed a capsaicin binding site within the membrane [5,6] distant to Asn604, regulation of ligand binding by extracellular glycosylation of Asn604 seems unlikely. Interestingly, Asn604 is absent from chicken TRPV1 that is insensitive towards activation by capsaicin. This work group also observed N-linked glycosylation of overexpressed TRPV2 [37] that is gated by heat, akin to TRPV1, but not by capsaicin. Although the glycosylation site of TRPV2 has not been mapped experimentally, Cohen conducted a sequence alignment of TRP segments that harbor known glycosylation sites and identified a conserved N-linked glycosylation motif near the pore-forming loop of TRPV2 [38]. Glycosylation adjacent to the pore-forming loop has also been shown for TRPV4 [39,40]. TRPV4 is strongly expressed in the distal convoluted tubules of the kidney and is gated by hypotonicity [53,54]. When Xu and co-workers treated TRPV4 from stably transfected HEK293 cells with endoglycosidase-F, an enzyme that removes N-linked oligosaccharides from proteins, they observed increased electrophoretic mobility of TRPV4 and thus inferred that TRPV4 is glycosylated [40]. In a follow-up publication, the authors identified Asn651 as the only possible N-linked glycosylation site that is situated on the extracellular side according to membrane topology prediction [39]. When Asn651 was mutated to Gln, they observed increased channel activity upon hypotonic stress. These results may suggest that glycosylation of extracellular loops affects channel gating. However, in the case of TRPV4, a larger portion of the mutated channel was located in the plasma membrane as compared to the wild type channel which may account for the observed increase in channel activity as assessed by fura-2 ratiometry [39]. In contrast to TRPV1, the TRPM8 ion channel is activated by low temperatures and cooling compounds such as menthol and icilin [55,56,57,58]. The TRPM8 glycosylation site was mapped to Asn934 within the pore region [25,26]. Ablation of this glycosylation site by exchange to Gln did not affect plasma membrane localization or multimerization [25,26] but resulted in a decrease of the response to cold and menthol [24]. Treatment of trigeminal sensory neurons with tunicamycin, a drug that inhibits N-glycosylation of proteins, mimicked the effect of ablation of the glycosylation site. Thus, the effect of the N-linked glycosylation on channel activity was shown both for heterologously expressed as well as for native TRPM8 channels [24]. TRPM4b was shown to undergo N-glycosylation at Asn988 (Asn992 in human TRPM4b) in the pore-forming loop [22]. Disruption of this N-glycosylation site resulted in faster disappearance of TRPM4b from the membrane pointing to a role of Asn988 glycosylation in stabilization of surface expression.

## 4. Covalent Modification of TRP Cysteine Residues

For decades, it has been assumed that a signaling molecule interacts with its receptors in a non-covalent manner by fitting into a receptor binding pocket because of its three dimensional structure. This is known as the lock and key model of receptor activation. In the recent past, this model has been challenged by the finding that odorant receptors recognize odorants rather by their molecular vibrations than by their shape [59,60]. This new model has become known as the swipe card model of odorant recognition [61]. Yet another mechanism of receptor activation involves reversible covalent modification of the receptor by its ligands, which have been described for several TRP channels.

### 4.1. Covalent Modification of TRPC5

One such covalent modification is protein S-nitrosylation that conveys redox-based signals of the cell. The signaling molecule, nitric oxide (NO), is covalently attached to the thiol group of a cysteine residue within the target protein. Since cellular Ca^2+^ and NO signals are widely used and coordinated with each other, Yoshida and colleagues sought to identify an abundantly-expressed Ca^2+^ channel that is regulated by NO [17]. By heterologous expression of a variety of TRPC and TRPM channels in HEK cells, they identified TRPC5 as the TRP channel exhibiting the strongest activation by NO donors such as S-nitroso-*N*-acetyl-DL-penicillamine (SNAP) and (*E*)-4-ethyl-2-[(*E*)-hydroxyimino]-5-nitro-3-hexenamide. When the membrane-impermeable agent 5,5’-dithiobis(2-nitrobenzoic acid) (DTNB) was administered, no rise of intracellular Ca^2+^ levels could be observed. However, providing DTNB from the intracellular side resulted in increased intracellular Ca^2+^ levels. Consistently, extracellular application of the membrane-permeable methanethiosulfonate derivative 2-amino ethyl methane thiosulfonate hydrobromide (MTSEA) but not of membrane-impermeable (2- (trimethyl ammonium) ethyl) methane thiosulonate bromide (MTSET) resulted in TRPC5 activation. The authors concluded that the cysteine residues that are modified by TRPC5 agonists are located at the intracellular side. To identify these residues, every single cysteine residue was exchanged by serine and the resulting mutant TRPC5 channels were tested for agonist sensitivity. As a result, mutation of Cys553 and Cys558 led to a drastic reduction of TRPC5 activity upon stimulation by agonists. These two residues are located N-terminal of the pore-forming loop and are conserved in other TRP channels of the TRPC and TRPV subfamilies. Interestingly, sensitivity towards protons and temperature was increased in TRPV1 upon application of SNAP [17]. To investigate the physiological role of NO-mediated TRPC5 activation in a native system, Yoshida and co-workers used bovine aortic endothelial cells that had been reported to express TRPC5 [62,63]. Native Ca^2+^ influx induced by NO donors was suppressed by expression of a dominant negative TRPC5 or by downregulation of TRPC5 via a siRNA approach [17] showing that TRPC5 considerably contributes to Ca^2+^ influx in this system. Since ATP had been shown to activate endothelial NO synthases (eNOS) via G protein-coupled receptor stimulation, the authors tried to reproduce this pathway in the endothelial cells. Application of ATP led to an increase in intracellular Ca^2+^ levels that was blocked by eNOS-targeted siRNA as well as by suppressors of S-nitrosylation. In aggregation, these results provide compelling evidence that S-nitrosylation is a post-translational modification regulating the activity of TRP channels in the context of NO signaling.

### 4.2. Covalent Modification of TRPA1

TRPA1 is expressed in the termini of nociceptive sensory neurons [64,65,66] and is activated by noxious exogenous compounds and endogenous proinflammatory mediators [10,67,68,69,70,71] resulting in cation influx and depolarization of the neuron that is ultimately perceived as pain by mammals [72]. Plants produce such pungent substances to protect themselves from herbivores. The pungent principal of the onion is allyl isothiocyanate (AITC), mustard produces mustard oil and cinnamon cinnamaldehyde, to name a few. As noticed in parallel by Hinman and colleagues as well as Macpherson and colleagues, most of these substances readily react with thiols and primary amines which led both groups to the assumption that these compounds covalently modify its receptor rather than activate it by specifically fitting into binding pockets in a lock and key manner [8,9]. To test their hypothesis, Hinman and colleagues made use of the synthetic electrophile cysteine-modifying agent N-methyl maleimide (NMM) because this compound irreversibly reacts with sulhydryl side chains under physiological conditions [8]. Indeed, whereas oocytes expressing human TRPA1 perfused with AITC showed a transient electrical response, application of NMM led to a persistent response. The NMM-induced response could be terminated by application of ruthenium red, a channel blocker, demonstrating channel specificity. By mutagenesis of candidate cysteine residues, the authors identified three cysteine residues, Cys619, Cys639, and Cys663 (Cys621, Cys641, and Cys665 in human TRPA1) residing within the predicted cytosolic N-terminus that confer sensitivity of TRPA1 towards electrophiles. The respective mutant TRPA1 exhibited strongly reduced sensitivity towards NMM and AITC but sensitivity towards δ-9-tetrahydrocannabinol, a non-electrophile agonist of TRPA1, was unaffected. This shows that the three identified cysteine residues specifically confer sensitivity towards electrophile substances and that non-electrophile substances gate this channel by another mechanism. Using click chemistry, Macpherson and co-workers showed that mustard oil and cinnamaldehyde derivatives covalently bound to murine TRPA1 [9]. The authors also demonstrated that the cysteine-modifying agents iodoacetamide (IAA) and MTSEA activate mouse TRPA1 expressed in HEK 293 cells. Mutation of Cys415, Cys422, and Cys622 (Cys414, Cys421, and Cys621 in human TRPA1) resulted in absence of TRPA1 stimulation by cinnamaldehyde and cold stimuli. Takahashi and co-workers reported that the human TRPA1 channel is activated by a variety of inflammatory mediators such as nitric oxide (NO), 15-deoxy-Δ^12,14^-prostaglandin J2 (15d-PGJ2), hydrogen peroxide (H_2_O_2_), and protons (H^+^) [10]. Site-directed mutagenesis of cytoplasmic N-terminal cysteine residues revealed that Cys421 and Cys621 mediate 15d-PGJ2 susceptibility of TRPA1.

Wang and colleagues identified four different disulfide bonds that are formed between five different cysteine residues *in vivo* [73]. These different constellations of disulfide bonds may result in different conformational modalities of TRPA1 that might provide different binding pockets for effector molecules as well as altered accessibility of cysteine residues for covalent modification.

Takahashi and colleagues uncovered a role of TRPA1 as an O_2_ sensor [11]. When the authors expressed a panel of redox-sensitive TRP channels in HEK 293 cells, they observed a rise in intracellular Ca^2+^ levels upon hyperoxia and hypoxia in cells that expressed TRPA1. TRPA1 exhibited hydroxylation at Pro394 that was removed upon hypoxia which led to channel activation. Current responses to hyperoxia were drastically reduced when Cys633 or Cys856 residues of TRPA1 were mutated. The authors proposed that under normoxia, TRPA1 is hydroxylated at Pro394 within the N-terminal ankyrin repeat by prolyl hydroxylases (PHDs) inactivating the channel. Under hypoxia, decreased O_2_ concentrations lower the activity of PHDs leading to elevated levels of active TRPA1 that is not modified at Pro394. Under hyperoxia, O_2_ directly oxidizes Cys633 or Cys856 or both. Oxidation overrides the inactivation by hydroxylation at Pro394 and activates the channel.

In *Drosophila*, TRPA1 is expressed in gustatory chemosensors to prevent the fly from taking up toxic electrophiles. The discovery of *Drosophila* TRPA1 and its role as a receptor of toxic electrophile compounds points to a common ancestral TRPA1 chemosensor for pungent substances. Thus, the detection of toxic electrophiles via covalent cysteine modification of TRPA1, which has the important biological function of prevention of intoxication, may be conserved from insects to man [74].

Collectively, current data point to a complex regulation of the TRPA1 channel by a large panel of substances some of which covalently bind to different cysteine residues. Formation of intramolecular disulfide bonds seems to influence TRPA1 conformation and might regulate the accessibility of other cysteine residues. In some cases, activation of TRPA1 rather depends on the chemical nature of agonists than on their three dimensional structure.

### 4.3. Covalent Modification of TRPV1

Besides TRPC5 and TRPA1, TRPV1 has been reported to undergo covalent cysteine modifications mediated by pungent compounds and by oxidative stress [30,31]. When the reports describing the activation of TRPA1 by pungent chemicals through covalent modification were published, the activation mechanism of TRPV1 by such substances was still controversial. Therefore, Salazar and co-workers set out to investigate TRPV1 activation by electrophiles [30]. To identify cells expressing TRPV1, they treated dissociated mouse dorsal root ganglia (DRG) neurons with capsaicin, the best-established agonist of TRPV1. In these cells, besides capsaicin, extracts from onion as well as garlic elicited considerable current increases. Allicin, a component of both onion and garlic, was identified as the active compound as allicin application elicited electrical responses of DRG neurons. Treatment with ruthenium red resulted in abolition of allicin-induced currents showing that these currents were mediated by a TRP channel. Application of DTT also resulted in robust suppression of these currents. Removal of allicin did not result in spontaneous reversal of the currents. The latter two observations led the authors to assume that activation of TRPV1 by allicin might be mediated by covalent modification akin to TRPA1 activation. To delineate the contribution of TRPA1 and TRPV1 to allicin-elicited responses, the authors used dissociated DRG neurons from *Trpv1^−/−^* and *Trpa1^−/−^* mice. Application of allicin induced action potentials and activated currents in DRG neurons from both *Trpv1^−/−^* mice expressing TRPA1 and *Trpa1^−/−^* mice expressing TRPV1. In a behavioral assay, the authors injected allicin into the paws of the *Trpv1^−/−^* and *Trpa1^−/−^* mice and their wild type littermates and determined the time the animals spent licking the injected paw. As a result, both *Trpv1^−/−^* and *Trpa1^−/−^* mice injected with allicin spent drastically more time licking their paws then control-injected animals indicating that TRPV1 or TRPA1 alone are able to elicit this behavior. Interestingly, the allicin-injected wild type littermates spent more time licking their paws than the single knockout mutants pointing to a synergistic effect of TRPV1 and TRPA1 in this scenario. In transfected HEK 293 cells expressing rat TRPV1, extracts from onion and garlic as well as allicin also induced currents that were reversed by DTT. Currents provoked by capsaicin, which does not modify cysteine residues, were not reversed by DTT. Pretreatment with allicin led to elevated capsaicin-induced currents. The cystein-reactive compound MTSEA elicited electrical responses in DRG neurons as well as in TRPV1-expressing HEK 293 cells. Pretreatment with MTSEA as well as MTSET led to elevated capsaicin-induced currents. Moreover, MTSEA resulted in an increased open probability of TRPV1 indicating that cysteine modification alone is sufficient to activate the channel. To identify cysteine residues that mediate sensitivity to cysteine-reactive compounds, Salazar and co-workers individually mutated the 18 cysteine residues of the rat TRPV1 primary sequence. Mutation of a single residue, Cys157 of rat TRPV1 (Cys158 in human TRPV1), residing in the predicted intracellular N-terminal region, resulted in insensitivity of the channel to MTSEA and garlic and onion extracts. Response to capsaicin, however, was not impaired. To provide further evidence that Cys157 is the only residue that is needed to confer TRPV1 sensitivity to cystein-reactive compounds, the authors generated a cysteineless TRPV1 mutant as well as a cysteineless TRPV1 in which only Cys157 was present. The cysteineless TRPV1 channel was functional and could still be activated by capsaicin but not by MTSEA or by onion and garlic extracts. In contrast, the cysteinless TRPV1 harboring Cys157 recovered activation by MTSEA and onion and garlic extracts that could be reversed by DTT. These experiments provide strong evidence for Cys157 as the only residue that confers sensitivity of TRPV1 to cysteine-reactive compounds.

Treating human TRPV1-expressing HEK293 cells with hydrogen peroxide (H_2_O_2_) that mimics oxidative injuries, Chuang and Lin observed a slow but robust increase of the currents elicited by capsaicin [31]. This sensitization of the channel could be reversed by strong reducing agents such as 2,3-dimercaptopropanol (BAL) or dithiothreitol (DTT) suggesting that H_2_O_2_-mediated oxidation of cysteine residues confer TRPV1 sensitization. To investigate the sidedness of cysteines involved in this process, the authors used membrane-impermeable dithio-bis-nitrobenzoic acid (DTNB) and membrane-permeable phenylarsine oxide (PAO) that both link adjacent thiol groups. Extracellular application of PAO but not DTNB elicited a TRPV1 response indicating the modulation-sensitive cysteines reside within the cell. These cysteine residues were then identified in a reversion mutagenesis approach using chicken TRPV1. At first, all cysteine residues were mutated and then single residues were reverted to Cys and the resulting TRPV1 channels were tested. As a result, Cys772 and Cys783 single revertants were identified as being amenable to PAO sensitization. Since these single revertants harbor only one Cys residue, the authors concluded that these Cys residues form disulfide bonds between different TRPV1 subunits. To identify Cys residues that form intra-subunit disulfide bonds, sets of Cys residues were reverted. As a result, a double revertant harboring Cys393 and Cys397 exhibited PAO sensitization. Co-expression of Cys393 and Cys397 single revertants did not restore PAO sensitivity suggesting intra-subunit formation of a disulfide bond. Notably, two Cys residues near the N-terminus and one Cys residue in the vicinity of the C-terminus mediated PAO-induced suppression of basal activity of chicken TRPV1. These residues are absent from mammalian TRPV1. Three of the four Cys residues that were found to mediate oxidative sensitization in chicken TRPV1 are conserved in mammalian TRPV1 (Cys387, Cys391, and Cys767 in human TRPV1). Since Cys158 (Cys157 in rat TRPV1) had been reported to be covalently modified by allicin [30], Chuang and Lin generated a quadruple mutant in which all four sites (Cys158, Cys387, Cys391, and Cys767) were mutated. The resulting channel was insensitive to H_2_O_2_ and PAO treatment. Triple mutants harboring mutations in Cys158, Cys387, and Cys767 or in Cys158, Cys391, and Cys767 were resistant to PAO. The authors concluded that upon oxidative treatment, Cys387 and Cys391 form an intra-subunit disulfide bond while Cys158 or Cys767 form disulfide bonds between different channel subunits. Upon prolonged or repeated exposure to capsaicin, sensory neurons become unresponsive to capsaicin due to a reduction of the Ca^2+^ influx [75], a phenomenon referred to as desensitization. Therefore, Chuang and Lin tested how oxidative treatment regulates desensitization. Upon oxidative treatment of TRPV1 that was desensitized by prolonged exposure to capsaicin, the authors observed strong sensitization. The other way around, pretreatment of TRPV1-expressing cells with PAO led to reduced desensitization by prolonged exposure to capsaicin. Finally, H_2_O_2_ treatment showed drastic synergism when applied in conjunction with phorbol di-butyrate that promotes phosphorylation of TRPV1 and low pH. Taken together, these results demonstrate that oxidative stress is a major signal to modulate TRPV1 channel activity that can override desensitization and augment previous stimuli.

## 5. Polyester Modification of TRPM8

A less common post-translational protein modification is the attachment of polyesters that are covalently bound to hydroxy groups of amino acid side chains and, in addition, form multiple hydrophobic interactions [76,77,78]. Recently, Cao and colleagues reported the identification of a poly-hydroxybutyrate (PHB) modification of mammalian TRPM8 by a mass spectrometry approach [27]. The authors found a large number of putative PHB modification sites within the N-terminus and in the S3-S4 linker region of TRPM8. Enzymatic removal of PHB and mutation of PHButylated serine residues as well as adjacent hydrophobic amino acids that might interact with PHB methyl groups led to TRPM8 channel inhibition. The authors concluded that PHB modification of TRPM8 is a prerequisite for its normal function.

## 6. Phosphorylation of Mammalian TRP Channels

Phosphorylation is an abundant reversible post-translational modification of proteins that is involved in regulation of a multitude of cellular processes. Protein phosphorylation is mediated by kinases that catalyze the addition of a phosphoryl group to a hydroxyl group of a serine, threonine, or tyrosine residue. All eukaryotic protein kinases share a common structure of the catalytic core domain and belong to the same superfamily. According to structural and functional properties, eukaryotic protein kinases are grouped into eight families. Protein dephosphorylation is mediated by phosphatases that catalyze the hydrolysis of phosphoryl groups. Exhibiting different catalytic mechanisms and structures, protein phosphatases are more heterogeneous than protein kinases. Protein phosphatases are divided into two groups, the serine/threonine protein phosphatases (STPs) and protein tyrosine phosphatases (PTPs). Whereas protein kinases preferentially phosphorylate their target proteins at certain residues that are flanked by a consensus sequence, protein phosphatases are believed to operate in a more unspecific manner. The phosphorylation level of a certain residue of a certain protein at a given time and physiological condition is the result of the activities of kinases and phosphatases that phosphorylate or dephosphorylate this residue. Thus, kinases and phosphatases have to be tightly regulated in order to generate certain phosphorylation patterns.

### 6.1. Phosphorylation of TRPC Channels

The members of the canonical TRP receptor subfamily, TRPC3, TRPC6, and TRPC7, are activated by the permeant diacyl glycerol (DAG) analogue, 1-oleoyl-2-acetyl-sn-glycerol (OAG) and this activation is reversed by the PKC activator 12-myristate 13-acetate (PMA) [19,79,80,81]. Trebak and colleagues showed that application of PMA indeed resulted in increased phosphorylation of TRPC3 *in vivo* [80]. By comparing TRPC3, TRPC6, and TRPC7 amino acid sequences, they identified conserved candidate PKC phosphorylation sites. Mutation of Ser712 to Ala resulted in abolition of PKC-mediated inhibition of TRPC3 [13]. The moonwalker mouse mutant exhibits cerebellar ataxia and abnormal Purkinje cell development [14]. This phenotype is caused by a point mutation of Thr635 (Thr646 in human TRPC3) that results in reduced PKC-mediated phosphorylation of TRPC3 in moonwalker mice. The mutated TRPC3 channel displays abnormal gating that leads to the death of purkinje cells. Kwan and colleagues observed cGMP-mediated inhibition of TRPC3 [15]. Mutation of two putative protein kinase G phosphorylation sites, Thr11 and Ser263, reduced cGMP-mediated channel inhibition. In contrast to the inhibitory effect of phosphorylation of TRPC3 by PKC and PKG, phosphorylation of TRPC3 by Src kinase is required for its activation by diacyl glycerol [16].

Zhu and co-workers observed activation of mouse TRPC5 by carbachol that activates muscarinic receptors [18]. However, application of carbachol resulted in rapid desensitization of TRPC5. This desensitization of TRPC5 was blocked by inhibitors of PKC. Mutation of candidate PKC phosphorylation sites led to identification of Thr972 (Thr970 in human TRPC5). Exchange of Thr972 to Ala resulted in a marked decrease of carbachol-mediated desensitization of TRPC5.

Akin to TRPC3 and TRPC5, TRPC6 is inhibited by PKC-mediated phosphorylation [19] but phosphorylation by the Src family protein kinase Fyn increases its activity [20]. TRPC6 phosphorylation by calmodulin-dependent kinase II (CaMKII) is a prerequisite for channel activation because inhibition of CaMKII prevented channel activation by carbachol [21].

### 6.2. Phosphorylation of TRPV1

Besides N-glycosylation and covalent modifications of cysteine residues, TRPV1 undergoes phosphorylation. Cesare and McNaughton observed that the heat-activated current in small cultured dorsal root ganglion neurons was sensitized by application of bradykinin. The sensitization by bradykinin was mimicked by activators of protein kinase C and prolonged by phosphatase inhibitors [82]. Although five PKC isoforms were present in sensory neurons, only PKCε translocated to the plasma membrane upon application of bradykinin. Constitutively active PKCε sensitized the heat response whereas bradykinin-induced sensitization was suppressed by a specific inhibitor of PKCε [83]. To delineate PKC-dependent TRPV1 phosphorylation sites that mediate sensitization, Numazaki and colleagues expressed rat TRPV1 in HEK293 cells and mutated predicted intracellular Ser and Thr residues to Ala [32]. Potentiation of capsaicin-induced currents by PMA and ATP was reduced when Ser502 or Ser800 (Ser502 and Ser801 in human TRPV1) were mutated. Ser502 resides in the first intracellular loop connecting transmembrane regions two and three and Ser800 is located in the intracellular C-terminal region. Consistently, phosphorylation of TRPV1 fragments harboring Ser502Ala or Ser800Ala was drastically reduced as compared to wild type fragments in an *in vitro* kinase assay using PKCε.

Because sensitization of TRPV1 by PKA had been proposed [84], Rathee and co-workers investigated the effects of PKA on heat-induced currents through TRPV1 and found that activation of the cAMP/PKA cascade by forskolin potentiated the heat-induced current in rat DRG neurons [34]. Mutation of three putative PKA phosphorylation sites, Thr144, Thr370, and Ser502 of the rat TRPV1 channel (Thr145, Thr371, and Ser502 in human TRPV1) and expression in HEK 293 cells resulted in strongly decreased forskolin potentiation of heat-induced currents [34]. Bhave and colleagues showed that activation of PKA led to phosphorylation of TRPV1 and prevented TRPV1 desensitization [33]. By mutagenesis of candidate phosphorylation sites, they identified Ser116 of rat TRPV1 (Ser117 in human TRPV1) as the site that mediates PKA-dependent blockage of desensitization. Collectively, these results propose that PKA phosphorylates TRPV1 at Ser145, Thr371, and Ser502 in human TRPV1 and thereby sensitizes the channel. Phosphorylation of Ser116 by PKA prevents desensitization of TRPV1.

Since Ca^2+^ influx into the nerve cell is the major outcome of TRPV1 stimulation, Docherty and co-workers tested the possibility of a Ca^2+^-mediated desensitization of TRPV1 [85]. Using rat DRG neurons, the authors found that desensitization of capsaicin-induced responses was reduced when extracellular Ca^2+^ concentrations were lowered. Moreover, desensitization was inhibited by a Ca^2+^ chelator and by a specific inhibitor of the Ca^2+^/calmodulin-dependent protein phosphatase 2B (calcineurin). Therefore, they proposed that the capsaicin-induced rise of intracellular Ca^2+^ levels activates phosphatase 2B that in turn dephosphorylates TRPV1 to promote desensitization. Testing a panel of kinases, Jung and colleagues found that only calmodulin-dependent kinase II (CaMKII) was able to render TRPV1 susceptible for activation by capsaicin after previous desensitization [36]. The authors concluded that phosphorylation of TRPV1 by CaMKII is a prerequisite for channel activation and mutated candidate CaMKII phosphorylation sites in rat TRPV1. They found that channel activity was abolished when two candidate CaMKII phosphorylation sites, Ser502 and Thr704 (Ser502 and Thr705 in human TRPV1), were ablated simultaneously. Mutation of putative PKA and PKC consensus motifs failed to disrupt capsaicin-induced channel activity. Activation of TRPV1 by acid was not impaired in the double mutant and did not exhibit desensitization in the wild type.

Jin and co-workers examined the role of the cellular tyrosine kinase c-Src in the modulation of rat TRPV1 [86]. They observed that capsaicin-induced currents through rat TRPV1 were abolished by the c-Src inhibitor PP2 and reduced when dominant-negative c-Src was co-expressed. Conversely, capsaicin-induced currents through rat TRPV1 were elevated by sodium orhovanadate, a tyrosine phosphatase inhibitor. Additionally Tyr-phosphorylated TRPV1 and Src kinase were shown by co-immunoprecipitation to interact. Zhang and colleagues showed that nerve growth factor (NGF) signaling ultimately led to activation of Src kinase. Src kinase phosphorylated TRPV1 at Tyr200 resulting in increased surface expression of TRPV1 [35]. The authors proposed that sensitization of TRPV1 by NGF can largely be explained by subcellular trafficking of TRPV1 to the plasma membrane that is induced by Scr kinase-dependent phosphorylation of TRPV1.

In general, phosphorylation sensitizes TRPV1 for activating stimuli whereas dephosphorylation renders it less susceptible for activation. Multiple kinases that are activated through different signaling pathways phosphorylate TRPV1 at different sites that exert different functions. Therefore, TRPV1 is not only activated by diverse ligands and heat but is also regulated through inputs from diverse signaling pathways. It is therefore believed that TRPV1 serves as a signal integrator combining different input signals that ultimately result in a certain Ca^2+^ level in the nociceptive cell.

### 6.3. Phosphorylation of TRPV4

The TRPV4 channel is activated by hypotonicity and is believed to function as an osmosensor in vertebrates [53,54]. Because tyrosine phosphorylation had been associated with hypotonic stress [87,88,89,90], Xu and colleagues used an anti-phosphotyrosine antibody to investigate tyrosine phosphorylation in immunoprecipitates from HEK293 cells stably transfected with V5-tagged TRPV4 [40]. Upon induction of hypotonic stress, they observed a transient increase of TRPV4 tyrosine phosphorylation. To confirm that TRPV4 tyrosine phosphorylation also occurred in a native system, endogenous TRPV4 was precipitated from a murine distal convoluted tubule cell line using an anti-peptide antibody that was raised against the C-terminus of murine TRPV4. Using the anti-phosphotyrosine antibody, the authors confirmed that Tyr phosphorylation of native TRPV4 was up-regulated by hypotonic stress. Treatment of TRPV4 overexpressing cells with PP1, a specific inhibitor of the Src family of kinases, led to diminished Tyr phosphorylation under hypotonic conditions in a dose-dependent manner. In contrast treatment with genistein, a general tyrosine kinase inhibitor, and with piceatannol, an inhibitor of the Syk protein-tyrosine kinase, did not significantly impair Tyr phosphorylation. By immuno­precipitation, TRPV4 was shown to physically interact with a panel of Src family tyrosine kinases. However, Lyn was the only Src family tyrosine kinase that displayed a hypotonicity- and time-dependent interaction with TRPV4. In support of this result, Lyn and V5-tagged TRPV4 colocalized in stably transfected HEK293 cells as shown by confocal microscopy using anti-Lyn and anti-V5 antibodies. Overexpression of wild type Lyn by transient transfection of HEK293 cells stably transfected with TRPV4 resulted in increased Tyr phosphorylation of TRPV4 as was assessed with a phosphotyrosine antibody after immunoprecipitation. Overexpression of dominant negative Lyn led to modest depression of TRPV4 Tyr phosphorylation. Next, the authors ablated several predicted TRPV4 phosphorylation sites by exchange to Phe and expressed the mutated V5-tagged mouse TRPV4 proteins in COS7 cells. Tyr phosphorylation was assessed using an anti-phosphotyrosine antibody. As a result, TRPV4 in which Tyr253 was changed to Phe displayed strongly reduced levels of Tyr phosphorylation. HEK293 cells expressing the Tyr253 mutant of TRPV4 did not exhibit hypotonicity-induced Ca^2+^ transients that were observed in HEK293 cells expressing wild type TRPV4. In aggregation, the authors identified a single phosphorylation site of TRPV4 that is phosphorylated by a Src family tyrosine kinase under hypotonicity which is a prerequisite for gating of the channel [40].

### 6.4. Phosphorylation of TRPM7

TRPM7 is ubiquitously expressed and interestingly, in conjunction to its function as a cation channel, displays serine/threonine kinase activity and autophosphorylation [23,91]. TRPM7 is activated by PLC-coupled receptor agonists such as thrombin, lysophosphatidic acid, and bradykinin [92] and regulates actomyosin contractibility by phosphorylating the C-terminus of the myosin IIA heavy chain [93]. Actomyosin contractibility has been associated with various cellular processes such as cell migration, shape, adhesion, and cytokinesis [94,95,96]. Phosphorylation of myosin IIA is promoted by massive autophosphorylation of the TRPM7 C-terminus at 46 autophosphorylation sites that are located N-terminal of the kinase domain. Abrogation of this highly phosphorylated region suppressed substrate phosphorylation but not kinase activity *per se*. These results suggest that autophosphorylation of the TRPM7 C-terminus promotes substrate recognition rather than kinase activity of TRPM7 [23].

## 7. Phosphorylation of *Drosophila* TRP and TRPL

### 7.1. Drosophila Phototransduction

The *Drosophila* phototransduction cascade is located within a specialized compartment of the photoreceptor cells in the compound eye, the rhabdomere. The rhabdomere is built by finger-shaped evaginations of the apical membrane, known as microvilli. Proteins of the phototransduction cascade are located within or at the cytoplasmic surface of the rhabdomeric membrane. A subset of these proteins is tethered by neither inactivation nor after potential (INAD) scaffolding protein. Interaction between INAD and its binding partners is mediated by PDZ domains (named after the first three proteins that were identified to harbor these domains, post synaptic density protein, *Drosophila* disc large tumor suppressor, zonula occludens-1 protein). INAD harbors five of these domains that consist of approximately 90 amino acids [97]. Through interaction of binding partners with two or more INAD molecules and through INAD-INAD interactions, a supramolecular network, called the INAD signaling complex, is formed [98]. Binding partners of INAD comprise phospholipase Cβ (PLCβ), eye-specific protein kinase C (ePKC), and the TRP ion channel [97,99,100,101,102]. Interestingly, in *Drosophila*, the TRP channel serves as an anchor that attaches INAD and the other INAD binding partners to the rhabdomeric membrane. In addition to TRP, a second light-activated channel, TRP-like (TRPL) is also present in the rhabdomeric membrane, but is probably not attached to the INAD signaling complex [97]. The phototransduction cascade is initiated by the absorption of a photon by the visual chromophore of rhodopsin, 11-*cis*-3-hydroxy retinal that is thereby isomerized to all-*trans*-3-hydroxy retinal [103]. This isomerization triggers a conformational change in the opsin apoprotein resulting in the activation of a heterotrimeric Gq protein. In the Gqα subunit, GDP is exchanged by GTP and the Gα subunit detaches from the Gβγ subunits and activates PLCβ. PLCβ, in turn, cleaves phosphatidyl-4,5-bisphosphate (PIP_2_), a building block of the rhabdomeric membrane, to yield soluble inositol-1,4,5-trisphosphate, diacyl glycerol (DAG) that stays in the membrane, and protons. It has been shown that the decrease of PIP_2_ and the increase of protons are necessary for finally activating the ion channels TRP and TRPL [104]. Recent work also shows that the cleavage of PIP_2_ results in a considerable change of the curvature of the rhabdomeric membrane as manifested by a light-triggered contraction of the entire rhabdomere that may open TRP and TRPL channels mechanically [105]. However, the exact mechanism of TRP and TRPL activation is still under debate. Activation of TRP and TRPL results in an influx of cations that depolarizes the photoreceptor cell.

### 7.2. Phosphorylation of TRP

Since ePKC is a member of the INAD signaling complex, it was speculated that this protein kinase might phosphorylate other members of this complex. Indeed, by *in vitro* kinase assays using immunoprecipitated signaling complexes and radioactively labeled ATP, ePKC was shown to phosphorylate INAD as well as TRP [106,107,108]. However, *in vitro* assays may not reproduce all aspects of the physiological conditions in the cell. The first identification of an *in vivo* phosphorylation site of the *Drosophila* TRP ion channel was reported by Popescu and colleagues [109] who unambiguously identified Ser982 as a TRP phosphorylation site in a mass spectrometry approach. In a *pkc* null mutant fly, the respective phosphopeptides could not be observed. The authors concluded that Ser982 is a phosphorylation site that is phosphorylated by ePKC *in vivo*. A transgenic fly that expressed a modified TRP channel in which the Ser982 phosphorylation site was ablated by exchange to Ala, displayed a prolonged deactivation of the photoresponse. However, this phenotype only became evident upon application of a very bright light stimulus. Notably, the *epkc* null mutant *inaC^P209^* displayed a prolonged deactivation of the photoresponse as well, although it did so under every light intensity tested.

**Figure 2:**
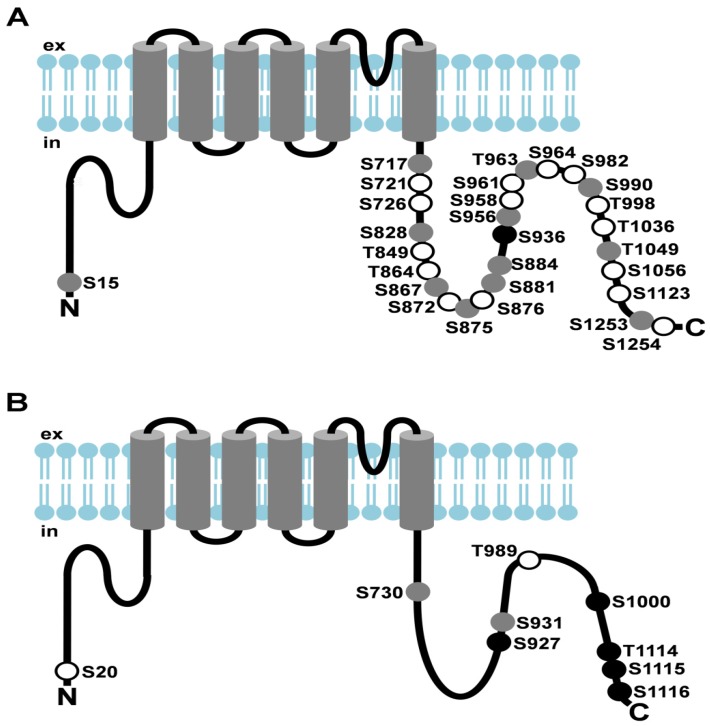
Light-dependent phosphorylation of *Drosophila* TRP and TRPL channels. (**A**) Cartoon depicting *Drosophila* TRP and its light-dependent phosphorylation sites; (**B**) Cartoon depicting *Drosophila* TRPL and its light-dependent phosphorylation sites. Amino acid residues that undergo phosphorylation are shown as circles. Sites that exhibited enhanced phosphorylation in the light are shown as white circles, sites that exhibited enhanced phosphorylation in the dark are shown as black circles, sites that revealed no significant difference in phosphorylation between light- or dark-adapted flies or that could not be assessed quantitatively are shown as grey circles; ex, extracellular; in, intracellular.

To identify TRP phosphorylation sites with a possible role in the regulation of vision, we analyzed TRP from light- and dark-adapted flies [42,43]. Using quantitative mass spectrometry, we were able to identify 28 phosphorylation sites, 27 of which resided in the predicted intracellular C-terminal region and a single site that resided near the N-terminus (Figure 2A). Fifteen of the C-terminal phosphorylation sites exhibited enhanced phosphorylation in the light, whereas a single site, Ser936, exhibited enhanced phosphorylation in the dark (Figure 2A). To further investigate TRP phosphorylation at light dependent phosphorylation sites, we generated phosphospecific antibodies that specifically detected TRP phosphorylation at Thr849, Thr864, which become phosphorylated in the light, and at Ser936, which becomes dephosphorylated in the light. We found TRP phosphorylated at Thr849, Thr864, and Ser936 to be located within the rhabdomeres indicating that phosphorylation of these sites is not a trigger for removal of TRP from the rhabdomeres. This finding further suggests that phosphorylation or dephosphorylation of TRP takes place at the rhabdomeres. To delineate the stage of the phototransduction cascade that is necessary to trigger dephosphorylation of Ser936 or phosphorylation of Thr849 and Th864, we exploited available *Drosophila* mutants of the phototransduction cascade. Flies were light- or dark-adapted and fly heads were subjected to Western blot analyses using the phosphospecific antibodies. We observed strong phosphorylation of Ser936 in dark adapted wild type flies but no phosphorylation in light-adapted wild type flies. Conversely, we found weak phosphorylation of Thr849 and Thr864 in dark adapted wild type flies and strong phosphorylation in light-adapted wild flies. These results were in accordance with our data obtained by LC-MS/MS. Additionally, we observed strong phosphorylation of Ser936 and weak phosphorylation of Thr849 and Thr864, regardless of the light conditions in mutants of the phototransduction cascade that exhibit impaired vision. In contrast, a mutant expressing a constitutively active TRP channel exhibited weak phosphorylation of Ser936 and strong phosphorylation of Thr849 and Thr864. These data indicate that *in vivo*, TRP dephosphorylation at Ser936 and phosphorylation at Thr849 and Thr864 depends on the phototransduction cascade but activation of the TRP channel is sufficient to trigger this process.

To identify kinases and phosphatases of Thr849 and Thr864, we conducted a candidate screen using available mutants of kinases and phosphatases that are expressed in the eye. We found that Thr849 phosphorylation was compromised in light-adapted *epkc* null mutants. Interestingly, we also found diminished phosphorylation in light-adapted *pkc53e* mutants suggesting that these two protein kinases C synergistically phosphorylate TRP at Thr849. Light-adapted *rolled* and *snf1a* mutants displayed significantly elevated phosphorylation levels of Thr849. Rolled is a mitogen-activated protein kinase that has been implicated in eye development [110,111]. The *snf1a* gene encodes an AMP-activated protein kinase. AMP-activated protein kinases have been associated to the cellular energy pathway [112]. Thr864 exhibited diminished phosphorylation in *ck1a*, *licorne*, *tao-1*, and *mppe* mutant flies. Neither of these mutations resulted a complete dephosphorylation of Thr864 showing that neither of these enzymes exclusively controls TRP phosphorylation at Thr864. Notably, MPPE is a metallophosphoesterase that mediates the activation of α-Man II that deglycosylates rhodopsin 1 in the process of its maturation [113]. 

### 7.3. Phosphorylation of TRPL

**Figure 3:**
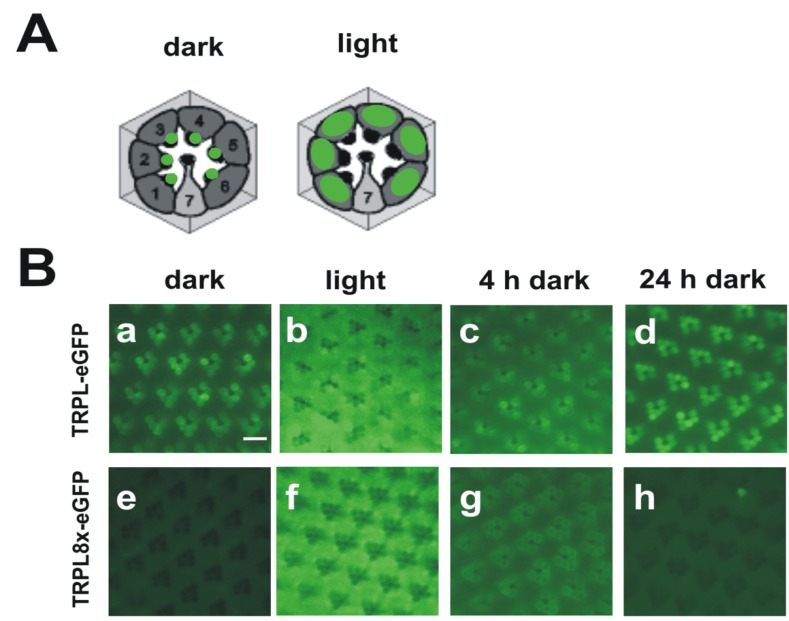
Light-dependent localization of *Drosophila* wild type TRPL and a TRPL channel lacking C-terminal phosphorylation sites (TRPL8x-eGFP). (**A**) Cartoons of cross sections through ommatidia showing localization of wild type TRPL in green. In dark-adapted flies, TRPL is located in the rhabdomeres but in light adapted flies, TRPL is located within the photoreceptor cell bodies; (**B**) Water immersion images of intact eyes of flies expressing TRPL-eGFP or TRPL8x-eGFP. In TRPL8x-eGFP the eight C-terminal phosphorylation sites were ablated by exchange to Ala. Flies were dark adapted for five days (dark) and were then light-adapted for 16 h (light). Subsequently, flies were dark adapted again for four hours (4 h dark) and 24 h (24 h dark), respectively. Scale bar, 10 µm. (Modified from [44]).

While the *Drosophila* TRP channel permanently resides within the rhabdomeric membrane, TRPL undergoes a light-dependent translocation from the rhabdomeric membrane to a storage compartment of yet elusive nature within the cell body [114]. Light-dependent translocation has been demonstrated for several phototransduction proteins such as Gqα and arrestin in *Drosophila* and is believed to function in long term light adaptation. Using mass spectrometry, we identified nine phosphorylated serine and threonine residues of the TRPL channel [44] (Figure 2B). Eight of these phosphorylation sites resided within the predicted cytosolic C-terminal region and a single site, Ser20, was located close to the TRPL N-terminus. Relative quantification revealed that Ser20 and Thr989 exhibited enhanced phosphorylation in the light, whereas Ser927, Ser1000, Ser1114, Thr1115, and Ser1116 exhibited enhanced phosphorylation in the dark. Phosphorylation of Ser730 and Ser931 was not light-dependent. To further investigate the function of the eight C-terminal phosphorylation sites, these serine and threonine residues were mutated either to alanine, eliminating phosphorylation (TRPL8x), or to aspartate, mimicking phosphorylation (TRPL8xD). The mutated TRPL channels were transgenically expressed in R1-6 photoreceptor cells of flies as *trpl*-*eGFP* fusion constructs. The mutated channels formed multimeres with wild type TRPL and produced electrophysiological responses when expressed in *trpl*;*trp* double mutant background indistinguishable from responses produced by wild-type TRPL. These findings indicated that TRPL channels devoid of their C-terminal phosphorylation sites form fully functional channels and they argue against a role of TRPL phosphorylation for channel gating or regulation of its biophysical properties. Since TRPL undergoes light-dependent translocation, we analyzed the subcellular localization of the phosphorylation-deficient as well as the phosphomimetic TRPL-eGFP fusion proteins by water immersion microscopy (see Figure 3 for TRPL8x-eGFP). After initial dark adaptation, wild type TRPL-eGFP was located in the rhabdomeres. After 16 h of light adaptation, TRPL-eGFP was translocated to the cell body and successively returned to the rhabdomeres within 24 h of dark adaptation. eGFP fluorescence obtained from the TRPL8x-eGFP displayed marked differences to the wild type. After initial dark adaptation, a faint eGFP signal was observed in the cell body but no eGFP signal was present in the rhabdomeres. After 16 h of light adaptation, a strong eGFP signal was observed in the cell body akin to that observed in the wild type. This indicated that TRPL8x-eGFP fusion construct was newly synthesized during light adaptation. After four hours of dark adaptation, TRPL8x-eGFP was present in the rhabdomere, but 20 h later, only faint eGFP fluorescence was observable in the cell bodies and none in the rhabdomeres.

Using immunocytochemistry, the eGFP signal of mutated TRPL in the dark was observed in restricted regions outside of the rhabdomere and differed from the diffuse eGFP signal observed in light-adapted flies expressing either the mutated or the native TRPL-eGFP channel. This result suggests that TRPL is localized in another subcellular compartment in dark-adapted TRPL8x-eGFP mutants that may be involved in degradation of TRPL. Unexpectedly, mutation of phosphorylation sites to Asp resulted in a similar phenotype as mutation to Ala comprising defects in TRPL localization and stabilization in the dark. This finding may be explained either by assuming that in this context, Asp did not mimic phosphate groups or that a specific pattern of TRPL phosphorylation—that changes upon exposure of the flies to light or darkness—is required for correct localization and stability of the channel. Taken together, our findings indicate that phosphorylation of TRPL at C-terminal residues seems to be required for retaining TRPL at its physiological site of action and thereby preventing degradation.

## 8. Concluding Remarks

The body of data about post-translational modifications of TRP channels is growing rapidly. However, some important questions remain unresolved. For many modifications it has not been thoroughly evaluated what fraction of the total amount of channels is modified at a given site. In the case of TRP phosphorylation, this fraction may range from a few percent to almost complete phosphorylation of all channel molecules. With regard to that, it would be important to find out whether a certain post-translational modification triggers a physiological response when present at a single subunit of a channel tetramer or only when this post-translational modification occurs at all four subunits at once. TRP regulation by modification of cysteine residues seems to be complex in some cases involving intra- and inter-subunit disulfide bonds that might influence the accessibility of cysteines by cysteine-modifying channel activators. In future work, the conditions under which certain disulfide bonds are formed have to be determined in greater detail and the physiological consequences have to be examined carefully. The physiological role of the massive C-terminal phosphorylation of the *Drosophila* TRP channel is still elusive. Generation of transgenic flies expressing modified TRP channels in which the phosphorylation sites are modified will help to shed light on this question. Furthermore, even if the physiological function of a certain post-translational modification is known, it is oftentimes not clear how that physiological function is achieved mechanistically. The mechanistic understanding of how post-translational modifications exert their physiological functions will be a major challenge in this research field.
